# Additive Manufacturing of SS316L/IN718 Bimetallic Structure via Laser Powder Bed Fusion

**DOI:** 10.3390/ma16196527

**Published:** 2023-10-01

**Authors:** Asif Mahmud, Nicolas Ayers, Thinh Huynh, Yongho Sohn

**Affiliations:** Department of Materials Science and Engineering, University of Central Florida, Orlando, FL 32816, USA; asif.mahmud@ucf.edu (A.M.); nicolas.ayers@ucf.edu (N.A.); thinh.huynh@ucf.edu (T.H.)

**Keywords:** laser powder bed fusion, bimetallic structure, constituent intermixing

## Abstract

Laser powder bed fusion (LPBF) is a popular additive manufacturing (AM) technique that has demonstrated the capability to produce sophisticated engineering components. This work reports the crack-free fabrication of an SS316L/IN718 bimetallic structure via LPBF, along with compositional redistribution, phase transformations and microstructural development, and nanohardness variations. Constituent intermixing after LPBF was quantitatively estimated using thermo-kinetic coefficients of mass transport and compared with the diffusivity of Ni in the austenitic Fe-Ni system.

## 1. Introduction

Additive manufacturing (AM) commonly refers to a process in which an engineering component is built in a layer-by-layer fashion close to their final (net) shape. This net-shape capability of manufacturing sophisticated parts with a high degree of accuracy from a computer-aided design (CAD) model and the potential for powder reuse, reduced cost, and reduced manufacturing steps make the AM a promising technology [1,2,3]. Laser powder bed fusion (LPBF) is a popular AM technique that can produce dense, complex parts by selectively melting the powder bed with a laser source in a layer-by-layer process [4,5,6]. Furthermore, stopping and restarting the process in LPBF has been observed to have minimal effects on the microstructure- and indentation-based mechanical properties of certain alloys [7]. This advantage of LPBF can be utilized to fabricate bimetallic structures (a structure consisting of two dissimilar metals and alloys). 

In the past, the joining of two metals/alloys was achieved through a variety of joining techniques, such as welding, brazing, or soldering. However, the metallurgical characteristics of traditional welding processes can result in an increased number of precipitates and intermetallics and the distortion of the weld interface, all of which are detrimental to the structural integrity of the final components. AM presents a viable alternative; it has the potential to avoid the complications found in traditional welding process and, most importantly, allows for the joining of metals/alloys without the use of any filler material [8].

Extreme environment applications such as in gas turbine engines, aerospace applications, or nuclear power generation require engineering components that must work at entirely different temperatures and at different locations or require site-specific properties throughout the component [9]. However, it can be challenging to leverage such characteristics from a single material, and this warrants the development of structural components with tailored properties. A bimetallic structure combines dissimilar alloys/metals with different densities and thermal and mechanical properties and has the potential to provide a solution to this challenging dilemma. To reduce the deadweight in automobiles, bimetallic materials have been suggested for use in the creation of body frames, engines, and pistons [10]. The applications of bimetallic structures range from reactors to heat exchangers in chemical industries, where some parts are required to be high-corrosion- and high-temperature-resistant (e.g., IN625 or IN718) and other parts are required to have good mechanical endurance/ductility (e.g., SS316L) [11]. These two alloys could provide promising opportunities in dissimilar material structures [12].

Hinojos et al. [8] reported the fabrication of IN718 onto a 316L stainless steel substrate and SS316L onto an Inconel substrate via electron beam melting (EBM); they also reported cracking at the interface of a SS316L deposited onto an Inconel substrate. Carroll et al. [9] reported a functionally graded material (FGM) of SS304L and IN625 fabricated via directed energy deposition (DED) and studied their microstructures, phase composition, and microhardness. Cracks were reported near the SS304 end of the gradient zone due to formation of a secondary phase. Locci et al. [13] studied the diffusion bonding and brazing of SS316L and IN718 for heat exchanger applications, which required survival for a prolonged period at around 560 °C. Hot press diffusion bonding revealed sound bonding with/without a pure nickel interlayer; however, fine porosity was observed at the interfaces and could be reduced with higher pressures and optimized bonding parameters. Ribeiro et al. [14] studied different interface mechanisms during the joining of multi-materials, and Stano et al. [15] reported an experimental study to improve the adhesion at the interface between stiff and soft materials via material extrusion (MEX) additive manufacturing technology.

Singh et al. [11] reported the fabrication of crack-free bimetallic SS316L on top of an IN718 structure via LPBF and observed good interface bonding. They reported an ultimate tensile strength (UTS) of 547.8 MPa and a ductility of 21.2% for the bimetallic structure. Ghanavati et al. [16] reported the fabrication of thin-walled FGMs of IN718 and SS316L via DED, which included the fabrication of three thin-walled FGMs (i.e., three layers of IN718 on top of three layers of SS316L). Sample 1 was fabricated with no gradient transition between the three layers of SS316L and three layers IN718; sample 2 consisted of two layers, with an equal weight percentage of base alloys sandwiched between the two layers of SS316L from the bottom and two layers of IN718 from the top. Sample 3 started with 100 wt% SS316L from the bottom, followed by a decrease of 20 wt% of SS316L in each subsequent step to form 100 wt% IN718 in the topmost layer. No defect was observed in sample 1, while sample 3 showed the greatest defects due to the significant amount of powder mixture in different proportions and different thermal behaviors of the base alloys. Mei et al. [17] investigated the fabrication of an SS316L/IN718/SS316L sandwich structure via SLM and reported an elasticity modulus of 103 ± 3 MPa, a ductility of 28.1 ± 2%, and a UTS of 596 ± 10 MPa, indicating good metallurgical bonding at the interfaces (i.e., reported interface width was approximately 100 μm) between the 316SS and IN718 alloys. 

This work focuses on fabricating SS316L/IN718 bimetallic structures with emphasis on the compositional redistribution, phase transformation, and microstructural development that takes place after LPBF to provide a fundamental understanding of the fabrication process. Constituent intermixing after LPBF was quantitatively estimated using thermo-kinetic coefficients of mass transport. The intermixing observed and quantified in the SS316L/IN718 bimetallic structures represents the intermixing of constituent alloying elements in all LPBF processes.

## 2. Materials and Methods

**Figure 1:**
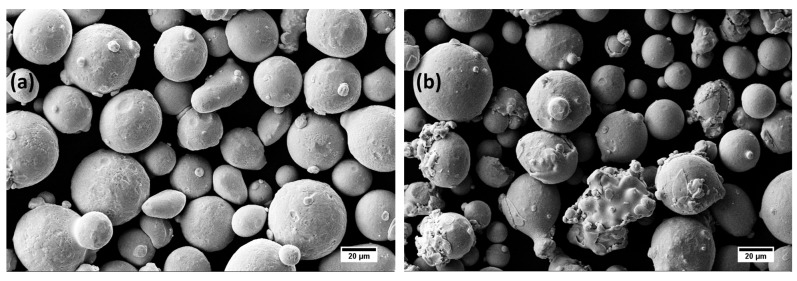
Secondary electron (SE) micrographs of virgin (**a**) SS316L and (**b**) IN718 powders.

Commercial SS316L and IN718 powders were acquired from SLM Solutions (SLM Solutions Group AG, Lübeck, Germany). The powder size distribution was examined using a laser diffraction particle size analyzer (Beckman Coulter LS^TM^ 13 320, Indianapolis, Indiana, USA). The powder particle sizes for the SS316L and IN718 were in the range of 19–46 µm and 18–49 µm, respectively. Chemical composition analysis of the powders was performed via X-ray energy-dispersive spectroscopy (XEDS) equipped with the field emission scanning electron microscope FE-SEM (FE-SEM, Zeiss Ultra-55TM; Zeiss USA, White Plains, NY, USA) and compared with the nominal compositions from the supplier. They are both listed in Table 1 and Table 2, respectively, for SS316L and IN718. The secondary electron micrographs of the virgin SS316L and IN718 powders employed in this study are presented in Figure 1.

**Figure 2:**
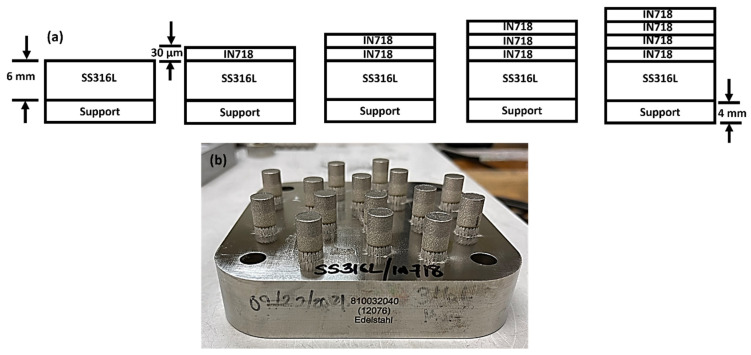
(**a**) Schematic of the layer-by-layer fabrication of the bimetallic structures via LPBF. A slice thickness of 30 μm was used to build both SS316 and IN718 to 6 mm in height. (**b**) Fabricated SS316L/IN718 bimetallic structures. The diameter and height of the cylinders were 10 mm and 12 mm, respectively.

For the LPBF of the SS316L/IN718 bimetallic structures, an SLM 125^HL^ (SLM Solutions Group AG, Lubeck, Germany) was utilized. The SLM 125^HL^ is equipped with a continuous-wave (1070 nm wavelength) 400-W Ytterbium IPG fiber laser with a Gaussian spot size of ~70 μm and a maximum build rate of 25 cm^3^/h [18]. Cylinders with a diameter of 10 mm and height of 12 mm, as presented in Figure 2, were manufactured using a laser power of 200 W, laser scan speed of 800 mm/s, hatch spacing of 0.12 mm, slice thickness of 0.03 mm, layer rotation of 45°, and stripe pattern of 10 mm width. Scanning strategies such as bordering or contouring were not employed. The cylinders were built onto an SS316L build plate for minimum thermal mismatch and good thermal conduction. The build plate was pre-heated to 100 °C. The build was performed in an inert Ar atmosphere (ultra-high purity Ar) with an O_2_ content lower than 0.10%. The SS316L/IN718 bimetallic structures were fabricated according to the following steps: (1) design the CAD file of the bimetallic structures, (2) employ the optimized processing parameters and transfer the *.stl* file to the LPBF machine, (3) fill the reservoir with virgin SS316L powders, (4) start the build using standard operating procedures, (5) build the SS316L cylinders on the SS316L build plate layer by layer for 200 layers until the desired height of 6 mm is reached, (6) pause the process, (7) remove the SS316L powders from powder feed reservoir and downpipe loader, (8) re-fill the reservoir with virgin IN718 powders and resume the process to complete the build. 

After the print, the samples were cross-sectioned using a low-speed saw. Each sectioned sample was mounted in epoxy and prepared using standard metallographic techniques. All samples were given a final polish using 1 µm diamond paste and colloidal silica (0.05 µm) polishing suspension. Once final polishing was completed, the bimetallic cross-section was examined using a Nikon Metaphot optical microscope to investigate the crack/pores near or at the interface. The SS316L/IN718 interface cross-section was then etched with mixed acids (HCl, acetic acid, and HNO_3_) with the volume ratio of 3:2:1.

A FE-SEM equipped with XEDS was used to examine the surface morphology, cross-sectional phase constituents, and microstructure of the interface cross-section. Both the secondary electrons (SE) and backscattered electron (BSE) imaging modes were utilized. A minimum of three concentration profiles across the interface of SS316L/IN718 were obtained via point-to-point acquisition to study the intermixing profile of the alloying constituents. The XEDS data were converted to the concentration of various constituent elements in atom percent via standardless analysis. X-ray diffraction (XRD) was carried out for phase identification and preferred crystallographic orientation analysis using a PANalytical Empyrean™ diffractometer with Cu target Kα radiation operating at 45 kV and 40 mA. A step size of 0.03° and a counting duration of 90 s were employed for a good resolution and statistical significance. 

Metallographically polished surfaces were examined for hardness measurements. Quasi-static nanoindentation was performed with a Berkovich tip using a Hysitron™ TI Premier Nanoindenter (Bruker, Minneapolis, MN, USA) on the specimens. Instrumented indentation was carried out at 5000 μN peak load with 10 s of loading time, 3 s of holding at the peak load, and 10 s of unloading. Hardness (H) and Reduced Modulus (*E_r_*) were determined by analyzing the unloading part of the load–displacement curve using Oliver and Pharr’s method [19,20]. Young’s modulus (*E*) was estimated from the reduced modulus (*E_r_*) using the following [19]:

(1) $$\frac{1}{E_{r}}=\frac{1-v^{2}}{E}+\frac{1-v_{i}^{2}}{E_{i}}$$

where *E_r_* is the reduced modulus of the specimen, *E_i_* is Young’s modulus of the diamond indenter tip (1140 GPa), *v* is the Poisson’s ratio of the specimen, and *v_i_* is the Poisson’s ratio of the diamond indenter tip (0.07). A total of 250 linear indents were performed with a spacing of 20 µm to determine the mechanical properties of the bimetallic interface via nanoindentation.

## 3. Results and Discussion

### 3.1. Phase Constituents and Microstructure

**Figure 3:**
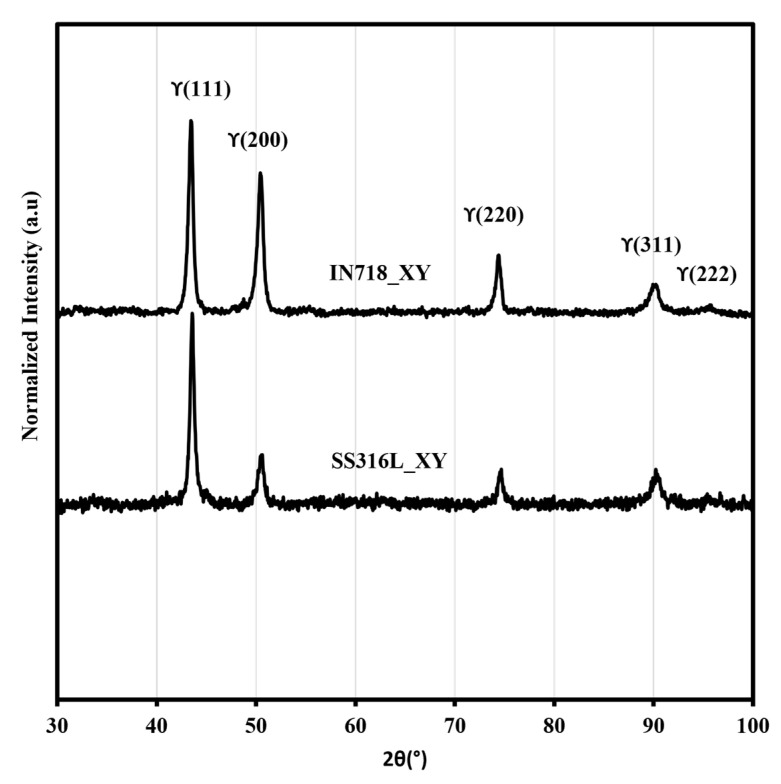
X-ray diffraction patterns of SS316L_XY and IN718_XY collected from the fabricated SS316L/IN718 bimetallic structures.

XRD patterns from the section normal to the build direction, XY (i.e., from SS316L and IN718), are presented in Figure 3. The main peaks observed from both the cross-sections corresponded to the austenitic *γ* (fcc) matrix, as shown in Figure 3. All the peaks were indexed via comparison with the powder diffraction files in PANalytical HighScore software. As the bimetallic interface region was observed to be less than 1 mm, the XRD pattern from this region did not yield any significant difference and is not included here. 

**Figure 4:**
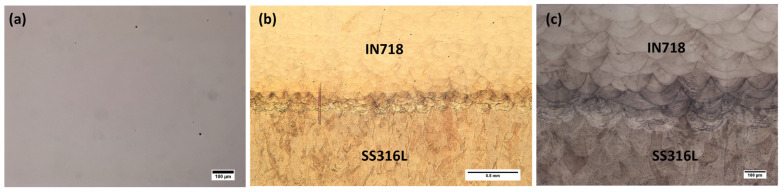
Optical micrographs of SS316L/IN718 interface cross-sections: (**a**) unetched, (**b**) low magnification etched and (**c**) high magnification etched.

No cracks were observed at the interface cross-section of the SS316L/IN718 bimetallic structures, and only a few pores were noticeable, as shown by the unetched optical micrograph in Figure 4a. The negligible porosity that can be observed at the interface region indicates sound metallurgical bonding between the two alloys and demonstrates that the bimetallic structures manufactured via the LPBF technique are nearly fully dense.

The low and high magnification etched cross-sectional optical micrographs at the interface region are presented in Figure 4b,c, respectively. The interface region consisted of well-developed semicircular overlapped SS316L and IN718 melt pools, and the interface width was observed to be approximately 200 µm. 

**Figure 5:**
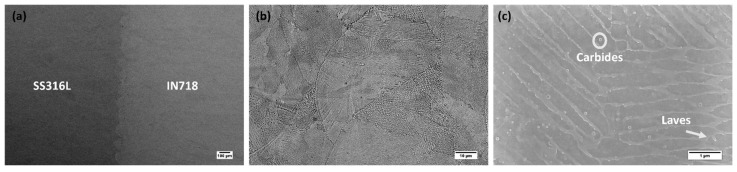
Electron micrographs of SS316L/IN718 bimetallic interface cross-section: (**a**) low-magnification BSE micrographs revealing the entire cross-section, (**b**) backscattered electron micrographs revealing the cellular/columnar microstructure of the interface, (**c**) high-magnification micrographs revealing the cellular/columnar microstructure along with the carbides and laves phases.

The scanning electron micrographs at high and low magnification in Figure 5 show the representative details of the microstructure and phase constituents at or near the SS316L/IN718 bimetallic interface. IN718 has a higher average atomic number, making it appear significantly brighter than SS316L, as presented in Figure 5a. The microstructure near the interface region of the SS316L/IN718 bimetallic structure consisted of typical cellular/columnar structure, as presented in Figure 5b. Also, the interdendritic region appeared with continuous white contrast compared to the dark contrast of the dendritic core, typically due to the segregation of heavier elements. Figure 5c revealed the presence of nanoscale Laves (indicated by arrows) and carbides (indicated by circles) near the interface of the SS316L/IN718 bimetallic structure, consistent with those reported by Mohd Yusuf et al. [21]. Typically, the LPBF of an as-fabricated IN718 alloy leads to a fine cellular or columnar dendritic microstructure resulting from the rapid cooling rate. In general, Nb, Mo, and Ti segregate along the cellular boundary and interdendritic region, resulting in the formation of nanoscale Laves and carbides. Precipitates of ϒ’ or ϒ’’ were not observed in the as-fabricated IN718 alloy. The formation of the Laves phase occurs through the eutectic reaction L → γ + Laves and is typically enriched with Nb [22,23].

### 3.2. Constituents Intermixing and Nanohardness

**Figure 6:**
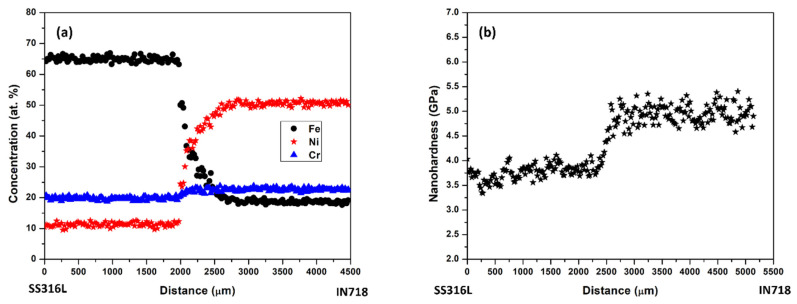
(**a**) Concentration profiles of Fe, Ni, and Cr; (**b**) nanohardness profile in the vicinity of SS316L/IN718 bimetallic interface.

Figure 6a shows the cross-sectional concentration profiles of Fe, Ni, and Cr across the SS316L/IN718 bimetallic interface, indicating the significant intermixing of Ni and Fe. No significant change in Cr concentration was observed because Cr concentration is similar for SS316L and IN718 alloys. Variation in laser power or scan speed can play a significant role in intermixing at the interface region. For example, a transition zone of approximately 3.5 mm was reported when IN625 was deposited upon SS316L fabricated on a mild steel substrate via DED by varying the laser power from 1.2 kW to 2 kW while keeping the other laser processing parameters constant [24].

In binary diffusion, typically, the chemical composition varies in the interdiffusion zone. Therefore, diffusing species continuously experience different chemical surroundings, and because the intermixing rate will depend on concentration, it will also vary throughout the intermixing zone. For such systems, Boltzmann [25] and Matano derived an integro-differential equation to calculate the interdiffusion at any given composition [26]:

(2) $$\tilde{D}=\frac{\frac{1}{2t}\int_{C_{i}^{\pm \infty }}^{C^{0}}\left(x-x_{0}\right)dC}{\frac{\partial C}{\partial x}}$$

where *C^+∞^* and *C^−∞^* refer to the composition at terminal ends of the diffusion couple, *x*_0_ is the position of the Matano plane, and *C*^0^ is the composition at the Matano plane.

The Matano plane denotes the location of mass balance such that:

(3) $$\int_{C^{+\infty }}^{C^{0}}xdC+\int_{C_{0}}^{C^{-\infty }}xdC=0$$

The intermixing fluxes of individual components, including the influence of convection, may be determined directly from their concentration profiles without the need for thermo-kinetic intermixing coefficients by using the following [27]:

(4) $$\tilde{J_{i}}=\frac{1}{2t}\int_{C_{i}^{\pm \infty }}^{C_{i}(x)}\left(x-x_{0}\right)dC_{i}(i=1,2,3\ldots \ldots n)$$

where *t* is the time. In the LPBF process, due to its small beam diameter (50–600 µm), the laser interacts with a small region (0.002 mm^2^–0.28 mm^2^) of the powder bed, resulting in a very high-power density (>10^4^ W/mm^2^) [28]. Such a high-power density yields an extremely high heating rate, followed by rapid cooling (10^4^ K/s–10^7^ K/s). The predicted time temperature relationship in a single laser study reported by Pantawane et al. [28] indicated varying cooling behavior within a temperature range of 3315 K–578 K in a time frame of 1 ms. Their result provides insight into the fact that the potential intermixing time for specimens produced via LPBF could be in the 1 ms range. 

To estimate the intermixing time for the specimens produced via LPBF, we used the concept of laser residence time. In LPBF, the laser residence time is the time of laser beam interaction at a particular location during scanning, and it is given by *t_r_ = d/v*, where *d* is the beam diameter; *v* is the scan speed of the laser. For the SS316L/IN718 bimetallic structures fabricated via the SLM 125^HL^ PBF unit, the beam diameter (spot size) was 70 µm (approximately), and the scan speed employed was 800 mm/s, which yielded a laser residence time of 875 µs for a layer thickness of 70 µm [29] (average melt pool depth was observed to be approximately 70 µm, with a laser power of 200 W and 800 mm/s). The estimated residence time is very close to 1 ms, the same as that reported by Pantawane et al. [28]. Significant intermixing of Fe and Ni was observed within 800 µm (intermixing zone) based on the concentration profiles presented in Figure 6a. So, the intermixing region basically went through a heat treatment (i.e., annealing) for a period of $\frac{800\;µm}{70\;µm}\times 875\;µs\;\sim$ 10 ms.

As the intermixing coefficient typically varies over the composition range, Dayananda [30] proposed an average effective interdiffusion coefficient that provides a single nominal thermo-kinetic coefficient for the compositional spectrum and allows for comparisons with other constant coefficients. Integrating the intermixing flux ($\tilde{J_{i}}$*)* over an interval from *x*_1_ to *x*_2_ and dividing by the change in composition over the interval, yields the following average effective interdiffusion coefficient:

(5) $$\tilde{D^{eff}=}\frac{\int_{x_{1}}^{x_{2}}\tilde{J_{i}}dx}{C_{x_{2}}-C_{x_{1}}}$$

**Figure 7:**
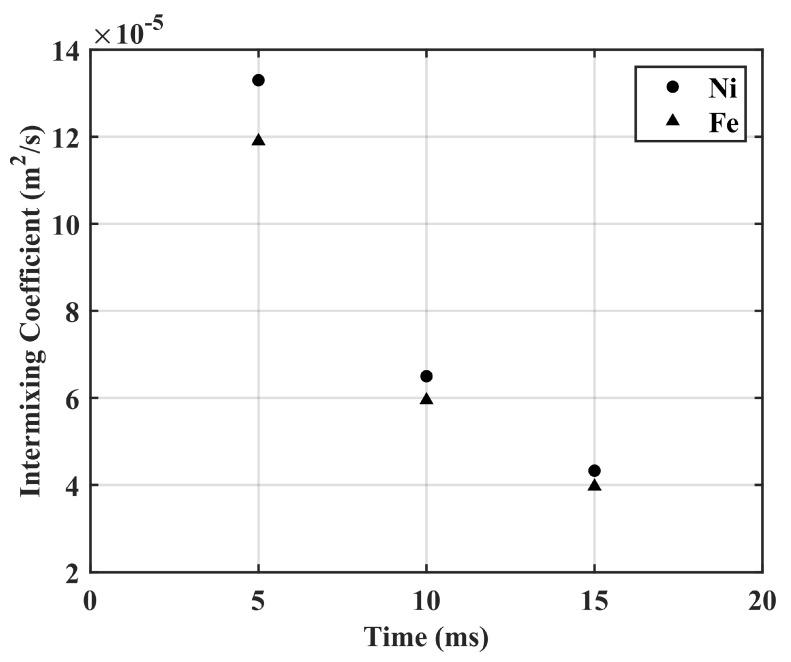
Intermixing coefficient of Ni and Fe as a function of time in SS316L/IN718 bimetallic structures.

This average effective interdiffusion coefficient was used to calculate the intermixing coefficient of Ni and Fe in the SS316L/IN718 bimetallic structures. The intermixing of the primary solvents Ni and Fe was observed for a diffusion zone of approximately 800 µm (Figure 6a), and the intermixing coefficient of Ni and Fe was estimated to be 6.5× 10^−5^ m^2^/s and 6.0 ×10^−5^ m^2^/s, respectively, based on a time of 10 ms. However, Figure 7 presents the intermixing coefficient of Ni and Fe as a function of time. In addition, the measured diffusivity of Ni in different solid-state Fe-Ni diffusion couples are presented and compared in Table 3 with the intermixing coefficient of Ni in the SS316L/IN718 bimetallic structure fabricated via LPBF. 

The mechanical properties of the SS316L/IN718 bimetallic structure derived from instrumented nanoindentation are presented in Figure 6b. In general, IN718 has higher hardness than SS316L, while the interface region was observed to have hardness in between the IN718 and SS316L structure. Typically, the nanohardness of additively manufactured SS316L ranges from 3.4 ± 0.69 GPa to 3.7 ± 0.64 GPa [31]. Zhou et al. [3] reported the nanohardness of as-built IN718 fabricated via LPBF to be 4.9 ± 0.13 GPa. Ghanavati et al. [32] reported a tensile strength of 581.72 MPa and ductility of 33.5% for a SS316L/IN718 bimetallic structure fabricated via LPBF.

**Table 3:** Diffusion coefficient of Ni in Fe-Ni diffusion couples and intermixing coefficient estimated in SS316L/IN718 bimetallic structures produced via LPBF.

Fabrication Technique	Terminal Composition (wt.%)	Temperature (°C)	Time (Days)	$\tilde{D}$ of Ni (m^2^/s)	Ref.
Diffusion Couple	Fe5Ni-Fe10Ni	910	~1	2.7 $\times \;10^{-18}$	[33]
	Fe5Ni-Fe10Ni	850	1.75	4.4 $\times \;10^{-19}$	[33]
	Fe10Ni-Fe15Ni	802	3	3.6 $\times \;10^{-19}$	[34]
	Fe10Ni-Fe15Ni	757	24	2.3 $\times \;10^{-20}$	[34]
	Fe15Ni-Fe20Ni	705	40	1.6 $\times \;10^{-20}$	[33]
	Fe20Ni-Fe25Ni	650	121	1.2 $\times \;10^{-21}$	[33]
	Fe25Ni-Fe30Ni	610	62	4.0 $\times \;10^{-22}$	[33]
LPBF	SS316L/IN718 Bimetallic	Varying	10 ms	6.5 $\times \;10^{-5}$	This study

## 4. Conclusions

This work reports on fully dense and crack-free fabrication of SS316L/IN718 bimetallic structures. The presence of negligible pores and nanohardness across the interface region between IN718 and SS316L indicates sound metallurgical bonding between the two alloys. The cross-sectional microstructure near the interface of the bimetallic structure consisted of a typical cellular/columnar structure. In addition, nanoscale Laves and carbides were also observed. The intermixing of primary solvents (Ni and Fe) was observed for a diffusion zone of approximately 800 µm, and their intermixing coefficient was estimated to be in the order of 10^−5^ m^2^/s.

## Figures and Tables

**Table 1:** Chemical composition (wt%) of SS316L powders.

	Fe	Cr	Ni	Mo	Mn	Si
Supplier	Bal.	16–18	10–14	2.0–3.0	2.0	1.0
XEDS	64.93 ± 0.33	18.86 ± 0.10	11.98 ± 0.15	2.21 ± 0.09	1.11 ± 0.22	0.91 ± 0.03

**Table 2:** Chemical composition (wt%) of IN718 powders.

	Fe	Cr	Ni	Nb	Mo	Co	Al	Mn	Ti	Si
Supplier	Bal.	17–21	50–55	4.75–5.50	2.8–3.30	1.0	0.20–0.80	0.35	0.65–1.15	0.35
XEDS	18.9 ± 0.59	20.4 ± 0.18	51.3 ± 0.59	4.6 ± 0.59	2.5 ± 0.21	0.1 ± 0.17	1.0 ± 0.2	0.1 ± 0.09	1.0 ± 0.16	0.2 ± 0.09

## Data Availability

Data are available from the corresponding author upon request.
